# Bloodletting Then and Now

**Published:** 1867-04

**Authors:** C. H. Spillman

**Affiliations:** Harrodsburg, Kentucky


*Bloodletting then and now.* By C. H. Spillman, M. D., Of Harrodsburg, Kentucky.

Fully persuaded that a proper conception of the *modus operandi* of bloodletting as a therapeutic agent, is an important *desideratum* in our profession. I propose to throw together, in as small a compass as may be, the results of inductions drawn from observation and experience, with special reference to this subject, running through a period of 35 years.

Believing, as I sincerely do, that the prevailing doctrines on this subject are unphilosophical, and lead to disastrous practical results, I read with much pleasure, Dr. Wilson's "Plea for the Lancet," in Vol. xv., No. 24 of the Reporter, as indicating a disposition on the part of the profession, to a more thorough examination of the subject, which, I doubt not, will lead to more rational views.

When I first came upon the arena in 1832, I was not long in becoming convinced that the lancet was used too indiscriminately, and sometimes to an injurious extent. I did not bleed as much as my neighbors, because I met with a number of cases that I could as easily and more safely control without than with it. In many others, however, it was a *sine qua non* to success.

How stands the matter now? Although diseases are the same, climate the same, morbific agencies the same; although organic structure is the same, vital susceptibilities the same, involving the same therapeutical relationships, pointing to the same indications of cure; yet such has been the revolution in the medical mind, that, at the present time, a large proportion of living practitioners rarely employ bloodletting as a remedial agent, and quite a number discard it altogether. Many of our late writers on therapeutics, if they justify its occasional employment, authorize it in such dubious phrase, with such admonitory qualifications and restrictions, as to clothe it in the garb of suspicion, and deter the junior members of the profession from its employment, even where indispensably called for.

I am not unaware of the fact, that the task before me is an ungracious one. It would have been more consonant to my feelings, could I have endorsed sentiments consecrated by so many justly distinguished advocates. To the popular doctrines on this subject, however, I find myself in a position of inexorable antagonism, by the logic of facts and figures which are impregnable. That more liberal enlightened views are demanded, and will ultimately obtain, I have an abiding conviction; and, whenever medical men shall have divested themselves of the leaven of empiricism, to which that distrust in regard to bloodletting as a remedial agent, which now sways the popular mind, may be legitimately traced, and come to view this subject in the light of rational physiological principles, we shall then have a fuller appreciation of that powerful remedy, which, as Dr. Wilson justly remarks, nature claims as her own, and shall have made an important step, toward the highest attainable perfectibility of our art.

From one opinion, however, advanced by Dr. W., in his excellent paper, I must take the liberty of dissenting. That this prejudice originated with the non-medical public, I think, is exceedingly questionable; and if the Dr. will submit the matter to a thoughtful review, he will find the responsibility where least excusable, with those who ought to know better. My observation is, that the popular verdict stands opposed to medical inefficiency in that regard. Disguise it as you may, I apprehend it is accepted as a concession to the various shades of empiricism which flood our land, all of which have been weighed in the balances and been found wanting.

There is no therapeutical agent, however valuable and indispensable to a successful exercise of our art, that may not be brought into disrepute by injudicious use; and a misapprehension which underlies the general prejudice which has obtained in regard to the lancet, relates to the principle on which it operates in the subversion of morbid action. Had it not been regarded as a physical agent, operating on mechanical principles, it would never have been confided to the hands of the ignorent, and we should have had fewer failures and miscarriages, which have contributed largely to this prejudice; for it is with this as it is with all other remedial agencies, the more powerful for good, the more prolific of evil, if misapplied. Employed as a vital agent, regardless of quantity, pushed to a given effect, by a practiced hand, under the guidance of a cultivated intellect, it is not only perfectly safe, but unquestionably the most potent remedial agent known to our art; nor can it be dispensed with, without surrendering to a weak vascillating timidity, compromising the most sacred obligations that can attach to a medical man, and greatly circumscribing the usefulness and efficiency of the medical art.

The argument against the lancet founded upon its supposed debilitating effects, is an abstraction, and not an induction from a careful observation of facts. On the contrary, every practitioner who has had an extensive experience in its employment, and witnessed its magical effect in the instantaneous subversion of the most violent forms of morbid action, appreciate it as a means of economising strength. In a sudden attack of either a congestive or inflammatory character, although the patient may have a feeling of great prostration, and is unable to put forth his strength, he is not weak. Take off the weight by which he is overborne for the time, and he is still strong.

"The giant," says an able writer, "that lies prostrate on the earth, mastered by superior power, has still a giant's strength, though he do not at that moment put it forth. Give him but the chance to throw off the load that keeps him down, and he will soon show you that he is not weak."

This is a very apt illustration of depressed vital action, misnamed debility, under the weight of disease. The intelligent physician will not be misled by the illusion. He will at once recognize this apparent debility as the sympathetic influence of a dangerous lesion in some vital part. Although greatly diversified in the phenomena they present, according to the peculiarity of the tissues involved, and the manifold remote causes which give rise to them, I apprehend there are few maladies not characterized by inflammation or venous congestion: either of which, by sympathetic influences, may occasion great prostration. They may be sudden in their onset; or insidious and gradual in their approach. They may persist for some time in a simple state of functional disturbance, but ofttimes run rapidly into irremediable structural alteration. In the latter case, relief if attainable, must be prompt and instantaneous. The practitioner, seeing the peril, and comprehending the situation, will find little room for temporizing. The most powerful means of equalizing the circulation and taking off the oppression, are called into requisition; and a philosophical view of the medium through which, and the manner in which, both morbific and remedial agents operate upon the vital economy, will at once suggest bloodletting as the most appropriate, because the most prompt and decisive means of accomplishing the object.

Irreconcilable as this may seem with that hypothesis, founded upon the mechanical philosophy, which assumes bloodletting to be a debilitent, it is nevertheless in strict accordance with the known therapeutical effect of that agent corroborated by the observation and experience of every one who has employed it, under an intelligent recognition of the principle on which it operates, in the subversion of morbid action.

The whole gist of the opposition to bloodletting, is predicated in conformity with the hypothesis, that it is necessarily debilitating; and this arises from a misconception of its *modus operandi* as a remedial agent.

Although a low pulse speedily raised, a shrivled surface filled out, cold extremities warmed up, equilibrium of circulation reinstated, lost strength restored, vital energy renovated, are phenomena which have been a thousand times observed to follow immediately on the intelligent employment of the lancet; and in multiplied instances such phenomena could have been elicited by no other means: it is nevertheless abandoned, on the ground of its alleged incompatibility with the assumed hypothesis.

Admitting the loss of blood to be intrinsically debilitating in a normal state of the system, and allowing our inability to reconcile this fact with its powerfully restorative effect in many forms of disease, the truth of which cannot be successfully controverted it is more seemingly paradoxical, than many well known facts with which the history of medicine abounds; and affords a striking exemplification of the practical value of the principle inculcated in Hoffman's Aphorism—"*Ars medica tota observationibus.*"

Quinia, in its nature and properties, is no less marked, intrinsically, as an excitant, than is the lancet a debilitant; and I apprehend the objector will find about as much difficulty in accounting, on philosophical principles, for the powerfully sedative influence of the former in controlling fever, as he will in reconciling the restorative influence of the latter, in diseases of depression, with its debilitating effects.

To assume an hypothesis on insufficient data, and then reject every principle not in harmony with it, is unphilosophical.

It is a humiliating fact, that in the present imperfect state of our knowledge, much of our reasoning, inconclusive, and unsatisfactory, rise no higher than mere speculation. However gratifying the reflection that by a close observance and careful analysis of facts, much is known in regard to the therapeutical effects of many remedial agents, still there are doubtless a great variety of hidden, unobserved influencing circumstances, connected with pathology, and therapeutics, which, if known, would greatly modify our inductions.

From a carefully noted and patiently classified series of facts, running through a long period, during which, in disregard of the popular prejudice, I have employed the lancet, not only to subdue violent inflammation, but to take off the oppression, and restore the strength, in cases of the most profound congestion, I am prepared to bear testimony to its magic power as a therapeutic agent; and hesitate not to say, after a patient persevering trial of all its reputed substitutes, that many such cases can be reached by no other means known to the profession.

The number of lives sacrificed to this prejudice against the lancet, I doubt not, is a thousand to one.

Look at the fearful increase of chronic diseases since the lancet has been partially ignored, and the profession has become tender-footed on the subject of bloodletting. Or to furnish a still more striking illustration: go to those districts where empiricism in its various forms, having manufactured, now subsists upon this prejudice, and lay it to the line and plummet of rigid vital statistics, and you will find multitudes of invalids who ought to have been restored to soundness by a prompt energetic treatment, whose cure, in consequence of an inefficient, temporizing course, has been incomplete;—vestiges of disease still remain;—vital lesion still lingers, ultimately to develope itself in some chronic form; and the tenure on life simply prolonged a brief period.

It is probable that tubercular disease in its diversified forms, is more destructive to human life than all other maladies combined. The best lights reflected from pathological anatomy note it as a product of inflammation. A patient investigation of the etiological history of very many of these diseases rarely fails to reach an inflammation as the point of inception. This fact is suggestive; and its inculcations should not be disregarded. It strikingly illustrates the folly of temporizing in all grave maladies; and affords the highest presumptive evidence against the expectant plan of treatment, which reposes upon the medical powers of nature, while disease, none the less destructive, from its insidious character, is stealthily settling down upon the vitals. The point is this, tubercular disease in its multiplied forms and various complications, is, in a large measure, the sequel to an inflammatory attack, which might and ought to be relieved by depletory measures so decisive, as to render the cure complete; and its great prevalence and fatality may be attributed to the existing popular prejudice against the only efficient means of subduing it in its incipiency.—*Reporter.*

